# Nitrate Capture Investigation in Plasma-Activated Water and Its Antifungal Effect on *Cryptococcus pseudolongus* Cells

**DOI:** 10.3390/ijms222312773

**Published:** 2021-11-26

**Authors:** Geon Joon Lee, Pradeep Lamichhane, Seong Jae Ahn, Seong Hwan Kim, Manesh Ashok Yewale, Choe Earn Choong, Min Jang, Eun Ha Choi

**Affiliations:** 1 Department of Electrical and Biological Physics, Plasma Bioscience Research Center, Kwangwoon University, Seoul 01897, Korea; theprodip@gmail.com (P.L.); maneshphd@gmail.com (M.A.Y.); 2 Department of Microbiology, Institute of Biodiversity, Dankook University, Cheonan 31116, Korea; asj0710@naver.com; 3 Department of Environmental Engineering, Plasma Bioscience Research Center, Kwangwoon University, Seoul 01897, Korea; cce_@live.com (C.E.C.); minjang@kw.ac.kr (M.J.)

**Keywords:** plasma-activated water, nitrate capture, Raman spectroscopy, *Cryptococcus pseudolongus*, cell viability

## Abstract

This research investigated the capture of nitrate by magnesium ions in plasma-activated water (PAW) and its antifungal effect on the cell viability of the newly emerged mushroom pathogen *Cryptococcus pseudolongus*. Optical emission spectra of the plasma jet exhibited several emission bands attributable to plasma-generated reactive oxygen and nitrogen species. The plasma was injected directly into deionized water (DW) with and without an immersed magnesium block. Plasma treatment of DW produced acidic PAW. However, plasma-activated magnesium water (PA-Mg-W) tended to be neutralized due to the reduction in plasma-generated hydrogen ions by electrons released from the zero-valent magnesium. Optical absorption and Raman spectra confirmed that nitrate ions were the dominant reactive species in the PAW and PA-Mg-W. Nitrate had a concentration-dependent antifungal effect on the tested fungal cells. We observed that the free nitrate content could be controlled to be lower in the PA-Mg-W than in the PAW due to the formation of nitrate salts by the magnesium ions. Although both the PAW and PA-Mg-W had antifungal effects on *C. pseudolongus*, their effectiveness differed, with cell viability higher in the PA-Mg-W than in the PAW. This study demonstrates that the antifungal effect of PAW could be manipulated using nitrate capture. The wide use of plasma therapy for problematic fungus control is challenging because fungi have rigid cell wall structures in different fungal groups.

## 1. Introduction

Recently, the effects of nonthermal atmospheric-pressure plasma on fungi, bacteria, and cancer cells have attracted considerable attention. An atmospheric-pressure plasma jet and dielectric-barrier-discharge plasma have been used to remove microorganisms, inactivate cells, increase plant growth, and treat cancer [1,2,3,4,5,6,7,8,9,10]. Plasma can generate reactive oxygen and nitrogen species (RONS). Interactions between plasma radicals and water molecules can produce additional reactive species [1,2] that might also be detrimental to fungi and bacteria. RONS-containing water produced by plasma treatment is called plasma-activated water (PAW) [1,2]. PAW is formed by the extraordinary exchange of energy and matter from plasma to water. Since the emergence of plasma medicine and plasma chemistry, PAW has attracted considerable interest for various applications in biological and medical science as well as in the water, agriculture, and food industries [1,2]. The mechanism of fungal spore inactivation by PAW is of particular interest because PAW can kill a larger volume of fungi than a direct plasma jet can [11,12]. To make PAW a practical tool, we need not only a fundamental understanding of its antifungal mechanism but also methods for handling the RONS in PAW in field applications. Generally, PAW becomes acidic because nitric acid forms from the plasma-induced RONS. When zero-valent magnesium (ZVM) is added to the water that is then treated with a plasma jet, the nitrate ions combine with the plasma-induced magnesium ions (${\mathrm{Mg}}^{2+}$). Electrons released from ZVM reduce the plasma-generated hydrogen ions into hydrogen atoms. The magnesium and nitrate ions exist in equilibrium with nitrate salt. The formation of nitrate salt raises the pH of the PAW and reduces the level of free nitrate ions. Consequently, it might be possible to use ${\mathrm{Mg}}^{2+}$ ions to control the level of nitrate in PAW. Lamichhane et al. demonstrated nitrogen fixation by metal ions produced in PAW using an atmospheric-pressure nitrogen plasma jet [13] and investigated the possibility of using nitrogen fixation for plant growth [14]. But no systematic study has performed the manipulation of nitrate ions in PAW generated using an atmospheric-pressure Ar plasma jet. It is beneficial to study the capture of nitrate ions in PAW by magnesium ions. Because using PAW in biological applications requires it to be safe and controlled, the development of an effective method to control the nitrate content has great merit in practical applications of PAW.

This study was performed to examine the magnesium ion-induced nitrate capture in PAW and its antifungal effect on the *Cryptococcus pseudolongus*. To our knowledge, this is the first report in which PAW exhibits an antifungal effect on a Basidiomycota fungus. This fungus has newly emerged as a mushroom pathogen that causes brown rot disease in shiitake [15]. In general, *Cryptococcus* is a genus of fungi that grow in culture as yeasts. A few species in the *Cryptococcus* genus cause a disease called cryptococcosis, which is a potentially fatal fungal infection of mainly the lungs and brain in humans [16]. To achieve our research aim, we measured the physicochemical properties of deionized water (DW) and Mg water treated with a plasma jet (PAW and PA-Mg-W, respectively), and we studied the capture of nitrate in PAW by plasma-induced ${\mathrm{Mg}}^{2+}$ ions. Next, we examined the effects of PAW and PA-Mg-W treatments on *C. pseudolongus* cells, and compared the viability of these PAW (PA-Mg-W)-treated cells with that of fungal cells treated by reference free nitrate (nitrate salt). Finally, we described variations in the viability of *C. pseudolongus* cells using the physicochemical properties of PAW and PA-Mg-W.

## 2. Results and Discussion

### 2.1. Electrical Characteristics of Atmospheric-Pressure Ar Plasma Discharge

**Figure 1:**
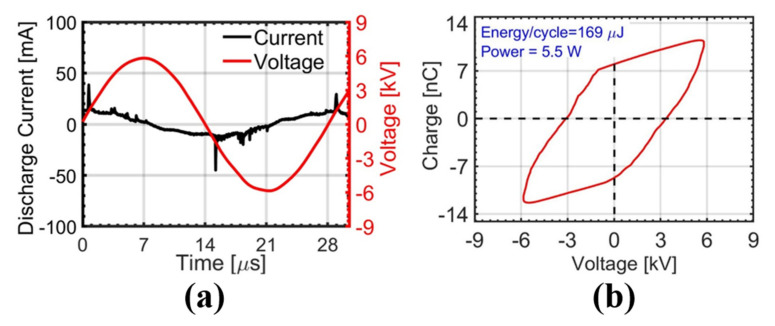
(**a**) Typical current and voltage profiles of the plasma discharge and (**b**) Lissajous plot corresponding to the plasma discharge.

Figure 1a shows typical current and voltage waveforms of the AC-driven plasma discharge. Several positive current peaks appear in the rising region of the applied voltage, and they result in the accumulation of charges inside a quartz tube. The measured discharge currents were a few tens of milliamperes with durations of a few tens of nanoseconds. During the plasma discharge, the reversed voltage changed the polarity of the accumulated charge. The corresponding charge (Q)-voltage (V) Lissajous plot is shown in Figure 1b. The Q-V plot becomes a closed loop, and its area gives the total energy dissipated in one cycle [17,18]. By fixing the area of the Lissajous plot to a constant value, the plasma treatments of both DW and Mg-W were performed under the same discharge power of 5.5 W [19]. This power corresponds to the energy per cycle of 169 μJ.

### 2.2. Analyses of Reactive Oxygen and Nitrogen Species Produced in Plasma Plume and Plasma-Activated Mg Water

**Figure 2:**
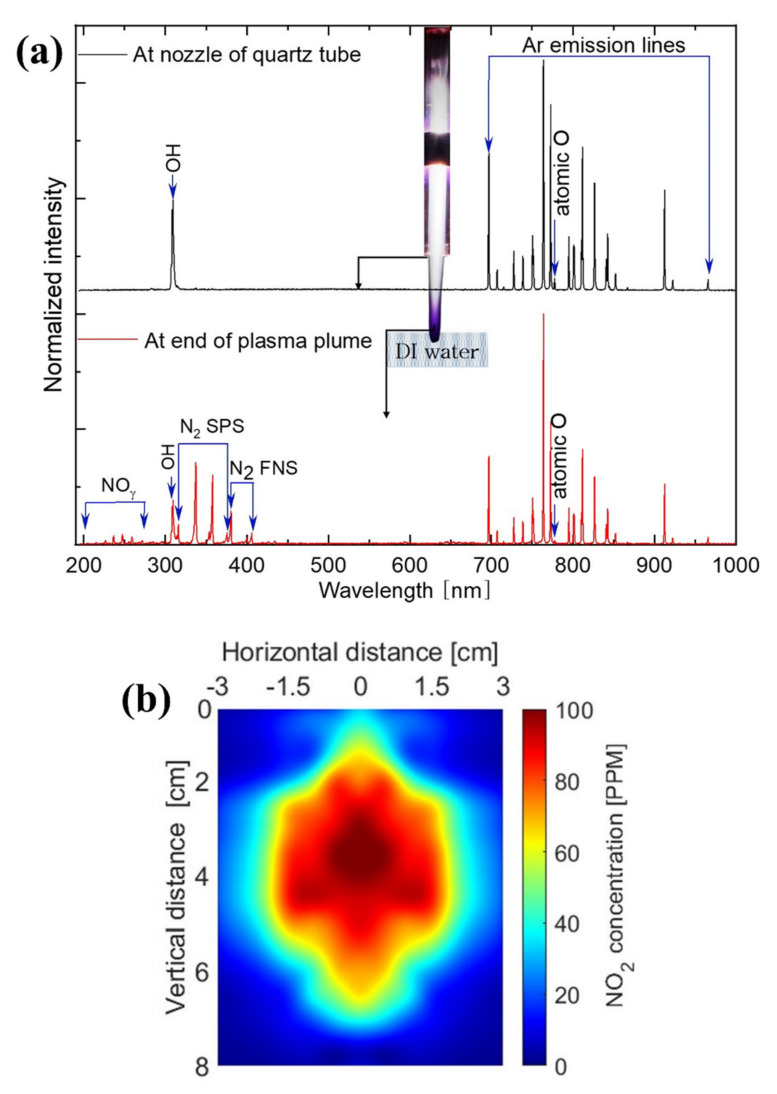
(**a**) Optical emission spectra of the plasma plume at the end of the quartz tube (black lines) and at the end of the plasma plume (red lines). (**b**) Longitudinal and transverse spatial distribution of ${\mathrm{NO}}_{2}$ for the plasma plume near the nozzle of the quartz tube.

To investigate the reactive species in the plasma jet, optical emission spectroscopy (OES) was used to measure the plasma plume at the end of the quartz tube and at the end of the plasma plume (a position 15 mm below the nozzle of the quartz tube). As shown in Figure 2a, the OES of the plasma plume at the end of the quartz tube mainly contained various reactive oxygen species (ROS), such as OH radicals and atomic oxygen. The emission lines of Ar were observed in the wavelength range of 690−925 nm because Ar served as the working gas, and our plasma device operated at atmospheric pressure [20,21]. The emission at 777 nm was produced by the 2s22p3S043s→2s22p3S043p transition of atomic oxygen [22,23]. The emission at 308 nm was generated by the AΣ2=XΠ2 transition of excited OH [24,25,26,27]. When oxygen molecules receive sufficient numbers of electrons via the plasma, they can be transformed into reactive oxygen intermediates (OH, $O_{2}^{-}$ and $H_{2}O_{2}$) through the $O_{2}$-reduction pathway [28,29]. Meanwhile, the OES at the end of the plasma plume showed various reactive nitrogen species (RNS) attributable to prominent molecular nitrogen lines of ${\mathrm{NO}}_{\gamma }$ [200–280 nm], the nitrogen second positive system [311–380 nm, N2CΠg3→BΠg3 transition)], and the first negative system [390–410 nm, N2+BΠu+2→XΠu+2 transition)] [25,26,27]. To confirm the plasma-induced RNS, we measured the spatial distribution of ${\mathrm{NO}}_{2}$ below the end of the quartz tube. The gas phase ${\mathrm{NO}}_{2}$ measurements were performed for the plasma jet propagating under ambient air without water. The longitudinal and transverse spatial distributions of ${\mathrm{NO}}_{2}$ are shown in Figure 2b. In the figure, the vertical distance represents the axial distance away from the end of the quartz tube along the propagation direction of plasma jet. The horizontal distance represents the radial distance away from the center of the plasma plume in the transverse plane perpendicular to the propagation direction of plasma jet. In the horizontal distribution, the negative sign represents the opposite side of the positive sign. In the PAW and PA-Mg-W experiments, the plasma jet is injected directly into water. The gas phase ${\mathrm{NO}}_{2}$ formed in the spatial region between the end of the quartz tube and the water surface dissolved into water. The ${\mathrm{NO}}_{2}$ distribution reveals that RNS were dominant at the end of the plume, whereas ROS were dominant at the end of the quartz tube.

**Figure 3:**
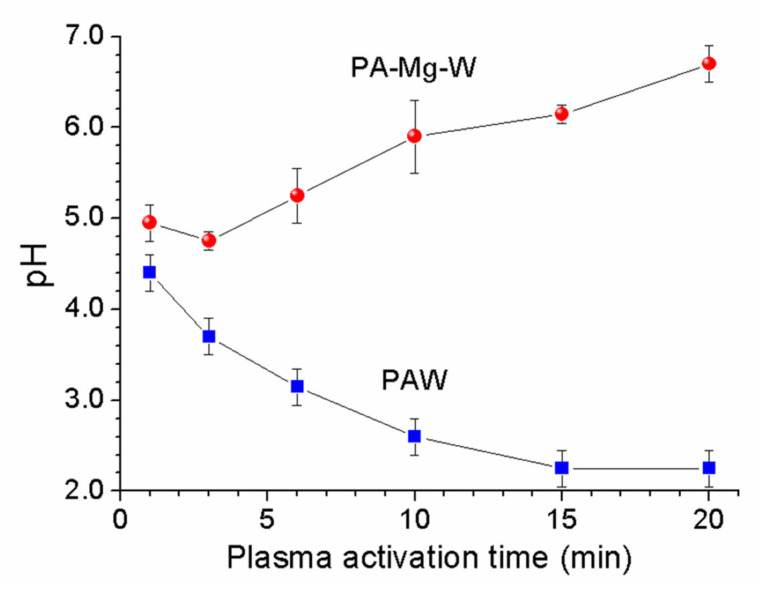
The pH of the PAW and PA-Mg-W as a function of the plasma activation time.

When a plasma jet enters an aqueous solution, additional reactive species could be generated from the interaction between plasma radicals and water molecules [20]. Reactive oxygen and nitrogen intermediates (OH, $O_{2}^{-}$, NO, ${\mathrm{NO}}_{2}$, and ${\mathrm{NO}}_{3}^{-}$) are converted into stable end products, hydrogen peroxide ($H_{2}O_{2}$) and nitric acid (${\mathrm{HNO}}_{3}$) [30,31], that can play key roles in deactivating microbial cells. As shown in Figure 3, the PAW was acidified by the nitric acid formed in water by the plasma jet, but the PA-Mg-W had a higher pH. In the PAW and PA-Mg-W, the possible nitrate generation and nitrate capture pathways were as follows [32,33,34,35,36,37,38,39,40,41,42,43]:

(1) $$\mathrm{Ar}+e^{-}\to {\mathrm{Ar}}^{*}+e^{-}$$

(2) $$O_{2}+{\mathrm{Ar}}^{*}\to O+O+\mathrm{Ar}$$

(3) $$N_{2}+{\mathrm{Ar}}^{*}\to N+N+\mathrm{Ar}$$

(4) $$N_{2}+{\mathrm{Ar}}^{*}\to N_{2}^{*}+\mathrm{Ar}$$

(5) $$H_{2}O+{\mathrm{Ar}}^{*}\to H^{+}+\mathrm{OH}+\mathrm{Ar}$$

(6) $$N_{2}^{*}+O\to \mathrm{NO}+N$$

(7) N+OH→NO+H

(8) N+O→NO

(9) $$\mathrm{NO}+O\to {\mathrm{NO}}_{2}$$

(10) $$\mathrm{NO}+\mathrm{OH}+M^{*}\to {\mathrm{HNO}}_{2}+M$$

(11) $${\mathrm{HNO}}_{2}+O\to \mathrm{OH}+{\mathrm{NO}}_{2}$$

(12) $$O_{2}+e^{-}\to O_{2}^{-}$$

(13) $$\mathrm{NO}+O_{2}^{-}⇌{\mathrm{NO}}_{3}^{-}$$

(14) $$H^{+}+O_{2}^{-}⇌{\mathrm{HO}}_{2}$$

(15) $${\mathrm{HO}}_{2}+{\mathrm{HO}}_{2}\to H_{2}O_{2}+O_{2}$$

(16) $$\mathrm{NO}+{\mathrm{HO}}_{2}+M^{*}\to {\mathrm{NO}}_{3}^{-}+H^{+}+M$$

(17) $${\mathrm{NO}}_{2}+\mathrm{OH}+M^{*}\to {\mathrm{NO}}_{3}^{-}+H^{+}+M$$

(18) $${\mathrm{NO}}_{3}^{-}+H^{+}\to {\mathrm{HNO}}_{3}$$

(19) $${\mathrm{Mg}}_{\left(s\right)}⇌{\mathrm{Mg}}_{\left(aq\right)}^{2+}+e_{\left(aq\right)}^{-}$$

(20) $$H^{+}+e_{\left(aq\right)}^{-}\to H$$

(21) $${\mathrm{Mg}}_{\left(s\right)}+2H^{+}\to {\mathrm{Mg}}_{\left(aq\right)}^{2+}+H_{2\left(g\right)}$$

(22) $$2{\mathrm{NO}}_{3}^{-}+{\mathrm{Mg}}_{\left(aq\right)}^{2+}⇌\mathrm{Mg}{\left({\mathrm{NO}}_{3}\right)}_{2}$$

where ${\mathrm{Ar}}^{*}$ denotes the metastable 4s state of argon; $M^{*}$ is an arbitrary reaction partner such as ${\mathrm{Ar}}^{*}$ or $N_{2}^{*}$; and $e^{-}$ and $e_{\left(aq\right)}^{-}$ are dry (nonhydrated) and wet (hydrated) electrons, respectively. In the plasma jet, N and O are generated by excited species from the dissociation of $O_{2}$ and $N_{2}$ (Equations (2) and (3)). NO and ${\mathrm{NO}}_{2}$ can be produced via various reaction pathways (Equations (6)−(11)). The main reaction leading to the generation of NO is Equation (7) [32]. Meanwhile, most ${\mathrm{NO}}_{2}$ is generated through the oxidation of NO (Equation (9)) [32]. NO and ${\mathrm{NO}}_{2}$ can be converted into ${\mathrm{NO}}_{3}^{-}$ ions (Equations (16) and (17)). In the PAW, water acidification was caused by plasma-generated hydrogen ions *(*$\mathrm{pH}=-\log_{10}\left[H^{+}\right]$) (Equation (5)). When ZVM was added to the water, magnesium dications (${\mathrm{Mg}}^{2+}$) were formed in the aqueous solution by plasma-generated hydrogen ions (Equation (21)), as shown in Figure 4. To detect the ${\mathrm{Mg}}^{2+}$ ion concentration in the PA-Mg-W, water containing a square disk-shaped Mg block (disk area = 1.0 cm×1.0 cm and disk height = 0.5 cm) was treated by the plasma jet for 10 min, and then the Mg block was removed. The resulting ${\mathrm{Mg}}^{2+}$ ion concentration was measured to be 80 mM by inductively coupled plasma (ICP)-OES. When the Mg block was immersed in water for 10 min without the plasma jet, the ${\mathrm{Mg}}^{2+}$ ion concentration in water was measured to be 0.26 mM. The formation of magnesium ions is facilitated in the acidic conditions caused by the plasma treatment. In the PA-Mg-W, ${\mathrm{Mg}}^{2+}$ ions can combine with the plasma-generated nitrate ions (Equation (22)). That is, free nitrate ions are converted into a stable nitrate salt, resulting in a reduction in free nitrate ions. The amount of formed nitrate salt depends on the number of ${\mathrm{Mg}}^{2+}$ ions. More ${\mathrm{Mg}}^{2+}$ ions can combine with more ${\mathrm{NO}}_{3}^{-}$ ions. The formation of nitrate salt will reduce the level of free nitrate ions, leading to a reduction in the antifungal effect by PAW. When nitrate salt is produced, ${\mathrm{HNO}}_{3}$ formation is suppressed (Equations (18) and (22)), and the PAW tends to be neutralized (Equations (20) and (22)) [13,44].

### 2.3. Optical Absorption Properties of Plasma-Activated Mg Water

**Figure 5:**
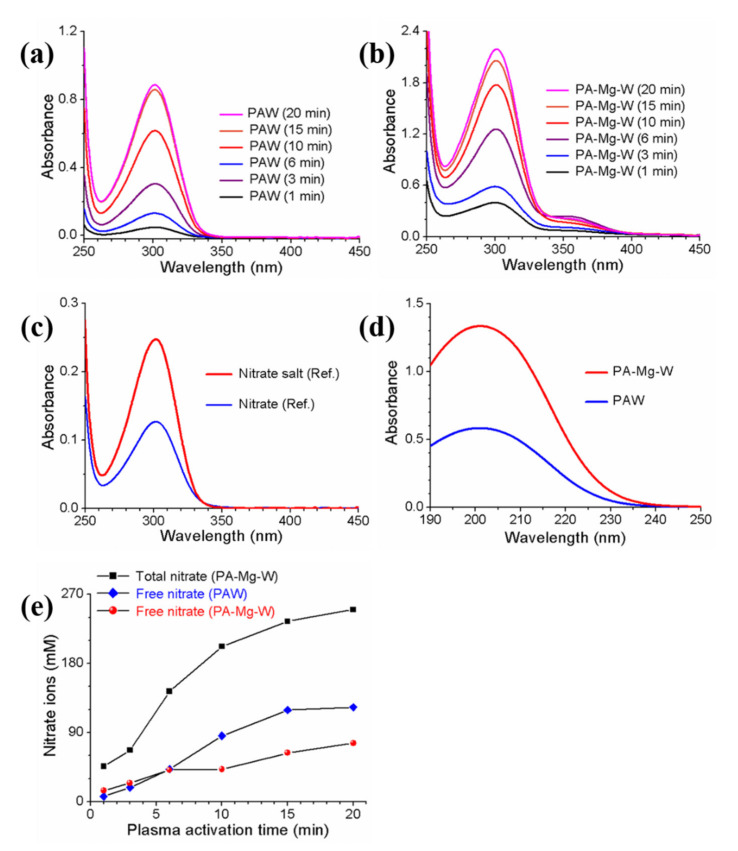
UV-visible absorption spectra of the (**a**) PAW and (**b**) PA-Mg-W over a wavelength range of 250–450 nm. (**c**) Absorption spectra of the reference free nitrate and nitrate salt ($\mathrm{Mg}{\left({\mathrm{NO}}_{3}\right)}_{2}\cdot 6H_{2}O$) solutions. (**d**) Absorption spectra of the PAW and PA-Mg-W over a wavelength range of 190–250 nm. (**e**) Nitrate ion concentrations in the PAW and PA-Mg-W.

The optical absorption spectra of the PAW and PA-Mg-W are shown in Figure 5a,b, respectively. The absorption spectra of the reference free nitrate and nitrate salt ($\mathrm{Mg}{\left({\mathrm{NO}}_{3}\right)}_{2}\cdot 6H_{2}O$) solutions are shown in Figure 5c. In this research, the commercial Oakton 1000 ppm nitrate standard was used as the reference free nitrate. The absorption spectra of the PAW and PA-Mg-W are similar to those of the reference nitrate and nitrate salt solutions. The DW was optically transparent in the UV and visible regions, but the absorption spectra of the PAW and PA-Mg-W exhibited a resonance absorption band at λ≃300 nm that is attributable to the nitrate ions produced in the PAW and PA-Mg-W by the plasma jet [45]. Longer plasma activation times produced higher concentrations of nitrate ions in the PAW and PA-Mg-W. To further investigate the plasma-induced nitrogen species, we diluted the PAW and PA-Mg-W and measured the optical absorption spectra over a wavelength range of 190−250 nm using a quartz cell with an optical path length of 2 mm. As shown in Figure 5d, the absorption spectra of the PAW and PA-Mg-W exhibited an absorption peak at 201 nm. As the reference nitrite and nitrate solutions have absorption peaks at 209 and 201 nm, respectively [12,46], the observed 201-nm absorption band indicates that nitrate ions greatly outnumber nitrite ions. Next, the nitrate absorption peak intensity at 300 nm was higher in the PA-Mg-W than in the PAW, as shown in Figure 5a,b. As calculated from the molar absorption coefficient of the reference nitrate solution, the nitrate concentration in the PAW treated by the plasma jet for 10 min was estimated to be 86 mM. When Mg water was treated by the plasma jet for 10 min, the free and bound nitrate concentrations in the PA-Mg-W were estimated to be 42 and 160 mM, respectively. The bound nitrate concentration in the PA-Mg-W was estimated from the ${\mathrm{Mg}}^{2+}$ ion concentration measured by ICP-OES (Figure 4), and the free nitrate concentration in the PA-Mg-W was obtained by subtracting the bound nitrate concentration from the total nitrate concentration. For PA-Mg-W and PAW with plasma treatment times between 10 and 20 min, the free nitrate concentration was lower in the PA-Mg-W than in the PAW (Figure 5e). These results indicate that plasma-generated ${\mathrm{Mg}}^{2+}$ ions can reduce the level of free nitrate ions in the PAW by forming nitrate salt. When DW and Mg-W were treated by the plasma jet for 10 min, the $H_{2}O_{2}$ concentration in the PAW and PA-Mg-W were estimated to be 98 µM and 88 µM, respectively, confirming that nitrate ions in the PAW and PA-Mg-W were the dominant reactive species that could affect the biological components of cells [47,48].

### 2.4. Raman Spectroscopic Study of Plasma-Activated Mg Water

**Figure 6:**
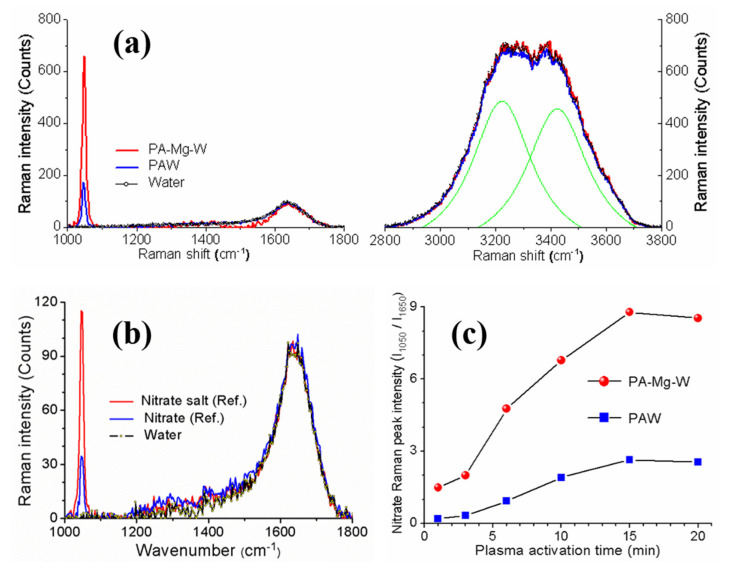
(**a**) Raman spectra of the PAW and PA-Mg-W. (**b**) Raman spectra of the reference free nitrate and nitrate salt solutions. (**c**) Nitrate Raman peak intensity as a function of plasma activation time. In (**a**), the Raman spectra were measured for PAW and PA-Mg-W treated by the plasma jet for 10 min.

Figure 6a shows the Raman spectra of the PAW and PA-Mg-W. The Raman spectra of the reference free nitrate and nitrate salt ($\mathrm{Mg}{\left({\mathrm{NO}}_{3}\right)}_{2}\cdot 6H_{2}O$) solutions are shown in Figure 6b. The Raman spectra of the PAW and PA-Mg-W exhibited several peaks attributable to specific vibration modes of water molecules and plasma-generated nitrate ions. As is well known, Raman bands in the frequency range of 3200 to 3600 ${\mathrm{cm}}^{-1}$ can be assigned to overlap the stretching vibration modes of water molecules at 3223 and 3422 ${\mathrm{cm}}^{-1}$, and the Raman band at 1630 ${\mathrm{cm}}^{-1}$ is due to the OH bending mode of water molecules [49,50]. The reference nitrate salt, PAW, and PA-Mg-W all exhibited an intense Raman band at 1049 ${\mathrm{cm}}^{-1}$, which is attributable to the symmetrical stretching vibration mode of nitrate ions [51,52,53]. Longer plasma activation times produced stronger Raman intensity because of the accumulation of plasma-induced nitrate ions. The 1049 cm^−1^ Raman band intensity is higher in the PA-Mg-W than in the PAW, as shown in Figure 6c, because the Raman scattering intensity of the bound nitrate ions in nitrate salt is stronger than that of free nitrate ions, as shown in Figure 6b. The Raman enhancement indicates that ${\mathrm{Mg}}^{2+}$ ions can convert free nitrate to bound nitrate by forming nitrate salt. To summarize, the Raman spectra of the PA-Mg-W and reference nitrate salt all exhibited a strong Raman peak near 1049 ${\mathrm{cm}}^{-1}$, indicating that ${\mathrm{Mg}}^{2+}$ and ${\mathrm{NO}}_{3}^{-}$ ions formed solvent-shared ion-pairs ($\mathrm{Mg}{\left({\mathrm{NO}}_{3}\right)}_{2}\cdot 6H_{2}O$) in the aqueous solution [53]. These results indicate that the intense Raman band at 1049 ${\mathrm{cm}}^{-1}$ is attributable to the capture of nitrate by ${\mathrm{Mg}}^{2+}$ ions. Therefore, the 1049 cm^−1^ Raman peak could be used as a fingerprint to confirm the capture of nitrate in PAW by magnesium ions.

### 2.5. Antifungal Effects of Plasma-Activated Water on C. pseudolongus Cells

**Figure 7:**
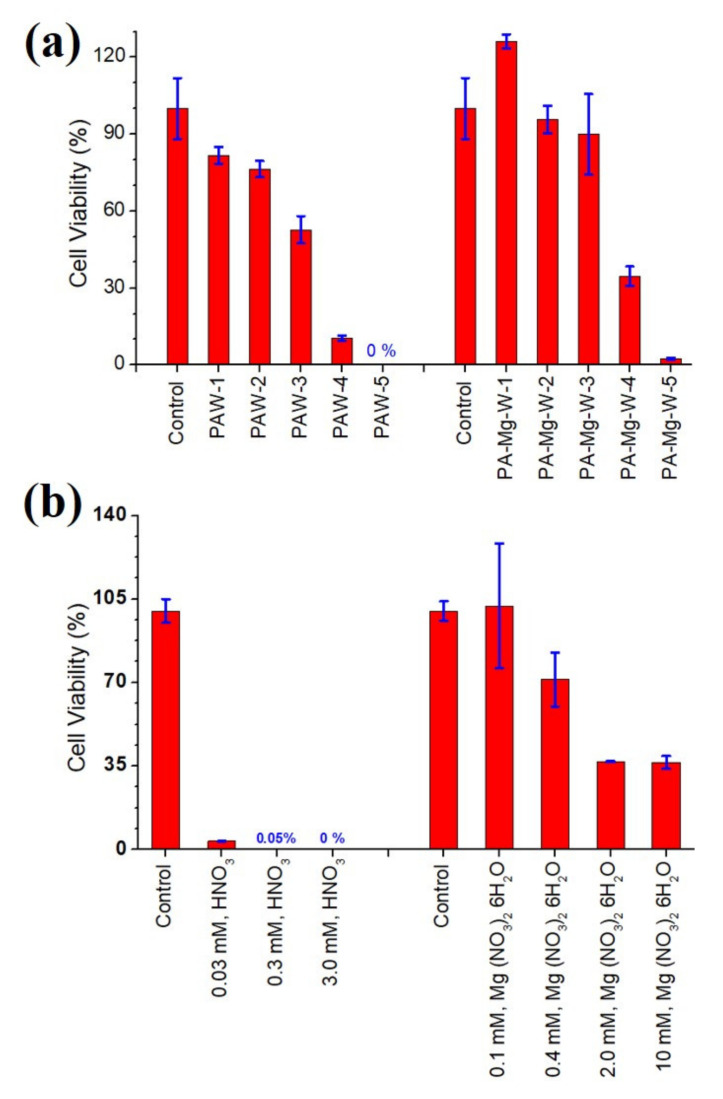
(**a**) Viability of *C. pseudolongus* cells treated with PAW and PA-Mg-W. The free nitrate ion concentrations were 0.14, 0.69, 3.4, 17, and 86 mM for PAW-1, PAW-2, PAW-3, PAW-4, and PAW-5, respectively. The nitrate salt concentrations were 0.26, 1.3, 6.3, 32, and 160 mM for PA-Mg-W-1, PA-Mg-W-2, PA-Mg-W-3, PA-Mg-W-4, and PA-Mg-W-5, respectively. (**b**) Viability of *C. pseudolongus* cells treated with nitric acid (${\mathrm{HNO}}_{3}$) and nitrate salt ($\mathrm{Mg}{\left({\mathrm{NO}}_{3}\right)}_{2}\cdot 6H_{2}O$ ) solutions. The fungal cells were in contact with PAW and reference nitric acid (nitrate salt) solutions for 24 h.

The cell walls of Ascomycota and Basidiomycota fungi contain chitin together with β-1,3, β-1,6-linked glucans in fibrous form. However, their structural compositions differ in the types of glycoprotein. That is, Ascomycota fungi are characterized by gel-like α-1,3 glucans and galactomannoproteins, whereas Basidiomycota fungi are characterized by α-1,3 glucans along with xylomannoproteins [54]. In a previous work, we demonstrated that PAW influences the spore viability and cell wall integrity of the Ascomycota fungus, *Aspergillus brasiliensis*, which has melanized fungal spores [12]. We wondered whether PAW would be applicable to Basidiomycota fungi. The cell wall of the basidiomycetous yeast *Cryptococcus* is enveloped by a gelatinous capsule composed of glucuronoxylomannan and galactoxylomannan that is anchored to the main wall via α-1,3 glucan. Therefore, we decided to test the antifungal effects of PAW on the Basidiomycota fungus. In addition, we investigated the possibility that the antifungal effects of PAW on the fungus could be manipulated through the capture of nitrate by magnesium ions. When testing antimicrobials, it is common to test at different concentrations to ensure that the effect appears in a concentration-dependent manner. Thus, we diluted the PAW and PA-Mg-W for the antifungal effect on the fungal cells. Both the PAW and PA-Mg-W treatments against *C. pseudolongus* cells revealed a remarkable effect on the viability of the fungal cells, as shown in Figure 7a. PAW-5 and PA-Mg-W-5 solutions were prepared by treating DW and Mg-W with a plasma jet for 10 min, respectively. PAW-1 (0.14 mM nitrate), PAW-2 (0.69 mM nitrate), PAW-3 (3.4 mM nitrate), and PAW-4 (17 mM nitrate) were prepared by diluting PAW-5 (86 mM nitrate) with DW. PA-Mg-W-1 (0.26 mM nitrate salt), PA-Mg-W-2 (1.3 mM nitrate salt), PA-Mg-W-3 (6.3 mM nitrate salt), and PA-Mg-W-4 (32 mM nitrate salt) were prepared by diluting PA-Mg-W-5 (160 mM nitrate salt) with DW. After being treated for 24 h in the PAW-5 solution, the fungal cell viability dropped to less than 1%, indicating that the PAW treatment effectively sterilized most of the tested *C. pseudolongus* cells. Longer plasma activation times lowered the fungal cell viabilities. The PAW-3 (3.4 mM nitrate) treatment exhibited cell viability of 53%. The PAW treatment reduced cell viability in a nitrate concentration-dependent manner (Figure 7a). The yeast type fungal cells are more sensitive in a low pH environment which could be established with a higher nitrate concentration (Figure 7b). The dilution of PAW with DW lowers free nitrate ion concentrations, makes the water less acidic, and leads the yeast cells to be less sensitive. Therefore, PAW can also destroy the cells of Basidiomycota fungi encapsulated by a relatively thick cell wall. These results demonstrated that the PAW produced by the atmospheric-pressure Ar plasma jet is detrimental not only to Ascomycota fungal spores which were produced by conidiophores [12] but also to Basidiomycota yeast cells which were produced by budding. From the viewpoint of applications such as sanitary means, it is meaningful that the two taxonomically different major groups of fungi are susceptible enough to the PAW produced by the plasma jet. Because nitrate ions were stable and long-lived reactive species and were confirmed to be present in the PAW from the optical absorption and Raman scattering spectra, we assume that the plasma-generated nitrate ions in the PAW were the key agents for the *C. pseudolongus* cell inactivation.

To provide evidence that nitrate ions can work against this fungal cell viability, we investigated the effect of a reference free nitrate treatment on the fungal cells. For this investigation, *C. pseudolongus* cells were treated with ${\mathrm{HNO}}_{3}$ solutions with nitrate concentrations similar to those in the PAW. As shown in Figure 7b, ${\mathrm{HNO}}_{3}$ treatments inactivated the *C. pseudolongus* cells. These results demonstrate that nitrate ions reduce the survival of fungal cells. Next, we found that the PA-Mg-W treatment also decreased the viability of fungal cells, as shown in Figure 7a. This result shows that the PA-Mg-W treatment also gives rise to an antifungal effect. When we compared the treatment effects of PAW and PA-Mg-W, *C. pseudolongus* cells had higher viability with the PA-Mg-W treatment. Therefore, the antifungal effect of PAW could be changed through nitrate capture. In fact, the viability of the PA-Mg-W-1 (0.26 mM nitrate salt)-treated cells were slightly higher than that of the control cells. Considering that nitrate can serve as a major source of nitrogen for most algae, bacteria, fungi, and higher plants and is the nutrient that most frequently limits their growth [55], we cannot rule out the possibility that the PA-Mg-W treatment at a particular concentration might enable cell growth of the fungus. However, further study is needed to verify that assumption. Nitrate salt ($\mathrm{Mg}{\left({\mathrm{NO}}_{3}\right)}_{2}\cdot 6H_{2}O$) solution treatments also showed higher cell viability than ${\mathrm{HNO}}_{3}$ solution treatments. The difference in viability between the PAW-treated cells and PA-Mg-W-treated cells is attributed to the decrease in free nitrate ions resulting from the formation of nitrate salts. Therefore, nitrate capture could attenuate the antifungal effect of PAW on *C. pseudolongus* cells.

## 3. Materials and Experimental Methods

### 3.1. Characterizations of the Atmospheric-Pressure Ar Plasma Jet and Plasma-Activated Mg Water

**Figure 8:**
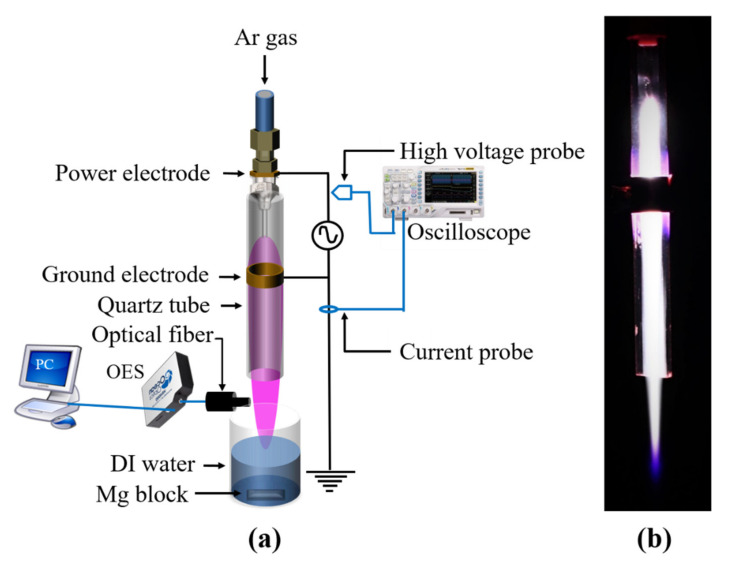
(**a**) Experimental setup of our atmospheric-pressure argon plasma jet system and (**b**) photograph of plasma plume. In (**a**), the plasma plume is pink-colored.

Figure 8 illustrates the experimental layout of our atmospheric-pressure Ar plasma jet and the photograph of a plasma plume. The plasma jet was created from a stainless steel needle electrode (length 35 mm, outer diameter 0.9 mm, and inner diameter 0.6 mm) inserted inside a quartz tube (length 55 mm, outer diameter 4 mm, and inner diameter 2 mm). The peripheral region between the top of the quartz tube and the needle electrode was sealed with a Torr seal. Argon gas entered the quartz tube through the opening of the needle. The flow rate of argon gas was continuously maintained at 1000 sccm. A specially designed DC-to-AC inverter with a repetition frequency of 33 kHz was used as the driving source to produce the plasma. For grounding, copper tape (width 3 mm) was wrapped around the quartz tube 5 mm below the lower tip of the power electrode. When an alternating sinusoidal voltage of ≅6 kV (peak-to-peak) was applied, plasma was produced between the two electrodes and then propagated along the quartz tube to reach air under ambient atmospheric pressure conditions. The plasma was then produced within the quartz tube below the lower tip of the needle. To obtain PAW in this experiment, the plasma was directly injected into the surface of 4 mL DI water with and without an immersed Mg block placed 25 mm away from the nozzle of the quartz tube.

The discharge voltage and discharge current were measured with a high-voltage probe (P6015A, Tektronix, Inc., Beaverton, OR, USA) and a current probe (CP030, LeCroy Corporation, Chestnut Ridge, NY, USA), respectively. The optical emission spectra of the plasma jet were measured using a fiber optic spectrometer (HR4000, Ocean Optics Inc., Dunedin, FL, USA). The spectrometer was wavelength-calibrated using a Hg-Ar lamp (6048, Newport Corporation, Irvine, CA, USA). To find the nitrate ion content in the PAW with and without Mg, UV-visible absorption spectra were measured using an optical spectrometer (J-815, Jasco Inc., Easton, MD, USA). Nitrate ion concentration in the PAW was obtained from the molar absorption coefficient of the reference free nitrate solution, and nitrate ion concentrations in the PA-Mg-W were obtained from the molar absorption coefficient of the reference nitrate salt ($\mathrm{Mg}{\left({\mathrm{NO}}_{3}\right)}_{2}\cdot 6H_{2}O$) solution. The concentrations of $H_{2}O_{2}$ in the PAW were measured using the QuantiChrom^TM^ Peroxide Assay Kit (DIOX-250) (BioAssay Systems, Hayward, CA, USA). To examine ${\mathrm{Mg}}^{2+}$ ions in the PA-Mg-W, total ${\mathrm{Mg}}^{2+}$ ion concentrations were measured via ICP-OES (Optima 2100 DV, Perkin-Elmer, Waltham, MA, USA) analyses after digestion in nitric acid following the 3500-Mg A standard method. To study the capture of nitrate in the PAW by magnesium ions, Raman spectra of the PAW and PA-Mg-W were measured at room temperature using a confocal Raman microscope (Alpha 300R, WiTec, Ulm, Germany) with a 632.8 nm He-Ne laser. To obtain the Raman spectra for the nitrate ions in the PAW, the incident laser beam was focused onto the PAW using a microscope objective (20×). The scattered light was collected by the same objective lens, dispersed by a grating, and then detected by a charge-coupled device array detector.

### 3.2. Fungal Growth and Antifungal Test of Plasma-Activated Mg Water

To test the antifungal effects of the PAW and PA-Mg-W, the mushroom pathogen *Cryptococcus pseudolongus* DUCC 4014 was used [15]. The antifungal test was performed against the yeast-like cells produced by this fungus. The fungus was maintained on potato dextrose agar (PDA; Thermo Fisher Scientific, Seoul, Korea) at 25 °C for 14 days. To prepare the cells, the fungal strain was grown in potato dextrose broth (Thermo Fisher Scientific, Seoul, Korea) on a shaker for 3 days at 25 °C. The cultured fungal broth was centrifuged at 8000 RPM for 5 min, the supernatant was removed, and the precipitated fungal cells were obtained. Sterile water was added to suspend the obtained fungal cells. The number of fungal cells was counted using a hemocytometer (Sigma-Aldrich, Bright-Line^TM^, St. Louis, MO, USA) and an optical microscope (Axioskop40, Carl Zeiss, Jena, Germany). Before PAW (PA-Mg-W) treatments, the concentration of fungal cells in water was adjusted to $5\times 10^{8}/\mathrm{mL}$. One volume of the prepared fungal cells was added to nine volumes of PAW (PA-Mg-W) so that the final concentration of fungal cells in PAW (PA-Mg-W) was $5\times 10^{7}/\mathrm{mL}$. After vortexing lightly, the fungal cells were treated with PAW (PA-Mg-W) for 24 h at room temperature. After centrifugation at 8000 RPM for 5 min, the supernatant was discarded, and the cells were dissolved with sterile DW. The dissolved fungal cells were diluted 10-fold with sterile DW, spread on PDA medium in three replicates, and incubated at 25 °C for 48 h, which was long enough to observe the surviving cells. The fungal colonies grown out on the plates were counted with a colony counter, and the number of colony-forming units was calculated to measure cell viability. To examine the effects of the reference free nitrate and nitrate salt solutions on the fungal cells, those solutions were prepared in different concentrations (0.03 mM, 0.3 mM, and 3.0 mM for the ${\mathrm{HNO}}_{3}$ solutions and 0.1 mM, 0.4 mM, 2.0 mM, and 10 mM for the $\mathrm{Mg}{\left({\mathrm{NO}}_{3}\right)}_{2}\cdot 6H_{2}O$ solutions). The fungal cells were treated with each concentration of ${\mathrm{HNO}}_{3}$ and $\mathrm{Mg}{\left({\mathrm{NO}}_{3}\right)}_{2}\cdot 6H_{2}O$ solutions for 24 h, as with the PAW and PA-Mg-W. Then the treated cells were centrifuged at 8000 rpm for 5 min, the supernatant was discarded, and the precipitated cells were dissolved with sterile DW. The dissolved fungal cells were analyzed for their viability as described for the PAW and PA-Mg-W treatments.

## 4. Conclusions

In conclusion, we demonstrated that the antifungal effects of PAW could be manipulated through the capture of nitrate by ${\mathrm{Mg}}^{2+}$ ions. Optical emission spectra of the plasma jet exhibited that ROS are dominant in the plasma plume at the end of the quartz tube, whereas RNS are dominant at the end of the plume, as shown by OES and the NO_2_ distribution of the plasma jet. The plasma treatment of DW produced acidified water. When Mg water was treated with the plasma jet, the acidity of the PA-Mg-W was weakened due to the reduction in the plasma-generated hydrogen ion by electrons released from the ZVM. Optical absorption and Raman spectra confirmed that nitrate ions were the dominant reactive species in the PAW and PA-Mg-W. Due to the formation of nitrate salts by ${\mathrm{Mg}}^{2+}$ ions, the free nitrate ion content was lower in the PA-Mg-W than in the PAW. The reduction in free nitrate ions in the PA-Mg-W is attributed to the formation of nitrate salts. Both the PAW and PA-Mg-W treatments against *C. pseudolongus* cells had a remarkable effect on the viability of the fungal cells. For PAW and PA-Mg-W treatments with the same plasma activation time, the viability of the fungal cells was higher in the PA-Mg-W than in the PAW. In parallel with the effort of development of new antifungal agents, there is increasing interest in the development of plasma devices to protect against the newly appeared fungal pathogens. From this work, we successfully extended the application of PAW from Ascomycota fungi to Basidiomycota fungi. This extension is also significant because PAW has effects on both yeast form and mycelial form cells which have different cell wall compositions. Consequently, we expect that PAW should be applied to antifungal therapies in various area in the very near future.

## Figures and Tables

**Figure 4:**
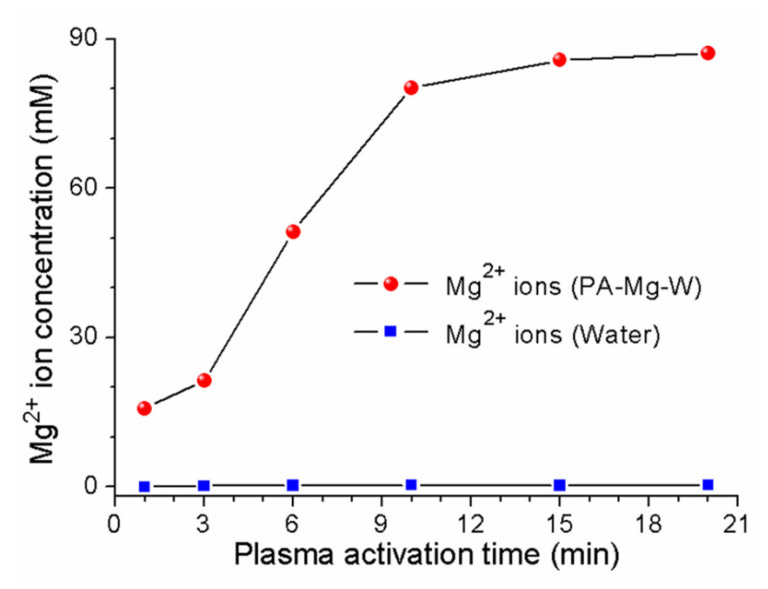
${\mathrm{Mg}}^{2+}$ ion concentrations in PA-Mg-W as a function of the plasma activation time. Red-colored circles and blue-colored squares represent ${\mathrm{Mg}}^{2+}$ ion concentrations when the Mg block was immersed in water for 10 min with and without the plasma jet, respectively, and then the Mg block was excluded from water.

## Data Availability

Not applicable.
